# Safety and efficacy of an herbal formula, *Gwakhyangjeonggi-san* on atopic dermatitis with gastrointestinal symptoms: Protocol for a randomized controlled trial

**DOI:** 10.1097/MD.0000000000020675

**Published:** 2020-07-10

**Authors:** Mi Ju Son, Min Hee Kim, Minseo Kang, Young-Eun Kim, Jeeyoun Jung, Inhwa Choi

**Affiliations:** a Clinical Medicine Division, Korea Institute of Oriental Medicine, Daejeon; b Department of Ophthalmology, Otolaryngology and Dermatology of Korean Medicine, Kyung Hee University Hospital at Gangdong, Seoul; c Future Medicine Division, Korea Institute of Oriental Medicine, Daejeon, Republic of Korea.

**Keywords:** atopic dermatitis, *Gwakhyangjeonggi-san*, herbal formula, *Huoxiang-zhengqi-san*, *Kakkoshoki-san*, randomized controlled trial

## Abstract

**Introduction:**

*Gwakhyangjeonggi-san* (GJS) is an herbal formula with anti-inflammatory and anti-allergic properties that is broadly used to treat a wide range of diseases including gastrointestinal disorders and allergic diseases. There have been several clinical studies conducted on its effects on atopic dermatitis (AD). So far, no randomized controlled trials have been conducted. Here, we describe the protocol for a randomized controlled study designed to investigate the efficacy and safety of GJS for treating patients with AD that have gastrointestinal symptoms.

**Methods and analysis:**

A randomized, double-blind, placebo-controlled, parallel-group, clinical trial has been designed to investigate the clinical efficacy and safety of GJS on patients with AD that have gastrointestinal symptoms. A total of 58 participants with AD will be recruited and randomly allocated to the GJS or placebo group in a 1:1 ratio. The participants will be administered GJS or placebo granules 3 times a day for 8 weeks. Data will be collected from the participants at baseline and after 4 and 8 weeks. The primary outcome measure will be the mean change in the SCORing of Atopic Dermatitis (SCORAD) index from baseline to 8 weeks. The secondary outcomes will include the eczema area and severity index (EASI), dermatology life quality index (DLQI), EuroQoL 5 dimensions 5 levels (EQ-5D-5L), and immunological factors. The Korean Gastrointestinal Symptom Rating Scale (KGSRS), Nepean Dyspepsia Index will also be obtained for assessing the gastrointestinal status.

**Discussion:**

The findings of this study are expected to provide evidence on the safety and effectiveness of GJS and for treating patients with AD that have gastrointestinal symptoms. Additionally, the study will explore the mechanism of GJS action via gut microbiome. This study will provide new perspectives on approaching treatment for AD.

**Ethics and dissemination:**

The study protocol was approved by the Institutional Review Board of Kyung Hee University Korean Medicine Hospital at Gangdong (KHNMCOH2019-06-002-001).

**Trial registration number:**

This study has been registered at the Korean National Clinical Trial Registry, Clinical Research Information Service (KCT0004299).

## 1 Introduction

Atopic dermatitis (AD) is a chronic, relapsing inflammatory skin disease that affects between 2.1% and 4.9% of adults and 20% of children around the world, but particularly in developed countries.^[1,2]^ AD is a complex and heterogeneous disease that is affected by various factors such as host genetics, altered skin barrier function, immunological abnormalities, and environmental factors, including exposure to specific pathogens.^[3]^

In recent years, gut microbiome has emerged as an important frontier in understanding the mechanism of AD.^[4,5]^ Recent evidence has demonstrated that dysbiosis of gut microbiota leads to a chronic progression of AD by dysregulation of gut inflammation and the gut epithelial barrier.^[6,7]^ Due to this evidence, it is expected that treatment that seeks to improve microbial composition is useful in the management of AD. Herbal drugs have been widely used for AD, based on their anti-inflammatory and anti-allergic properties.^[8]^ Recent studies have also shown the effects of herbal medicine on gut microbiota, which act as prebiotics to promote the growth of some gut microbiota species.^[9]^

*Gwakhyangjeonggi-san* (GJS), also known as *Huoxiang-zhengqi-san* in Chinese and *Kkako-shoki-san* in Japanese, is an herbal drug approved by the Ministry of Food and Drug Safety (MFDS) of the Republic of Korea. It is commercially marketed for anorexia, diarrhea, and fatigue^[10]^ and is used to treat a wide range of disorders, including gastrointestinal conditions^[11]^ and allergic diseases.^[12]^ GJS has demonstrated anti-inflammatory and anti-allergic activities in in vitro^[13]^ and in vivo studies.^[14]^ GJS was seen to improve AD symptoms and regulate serum cytokine levels in a clinical study.^[15]^ Moreover, GJS-probiotic combination treatment has been shown to be beneficial to gut microbiota counts.^[16]^ However, no randomized controlled trial has been conducted to report the efficacy and safety of GJS for AD in the view of the gut-skin axis.

Based on these previous studies, we hypothesize that GJS will improve the symptoms of AD by affecting gut microbiota, its related metabolites, and the immune system. This study aimed to observe the efficacy and safety of GJS in patients with moderate AD and gastrointestinal symptoms. Additionally, we will investigate the mechanism of the GJS action by analyzing immune biomarkers as well as microbiome in a randomized controlled experiment.

## 2 Methods

### 2.1 Objective

This protocol will be illustrated clinical trial design to investigate the efficacy and safety of GJS in patients with moderate AD concomitant with gastrointestinal symptoms and verify its mechanism via gut microbiome and immune biomarkers.

### 2.2 Study design

**Figure 1:**
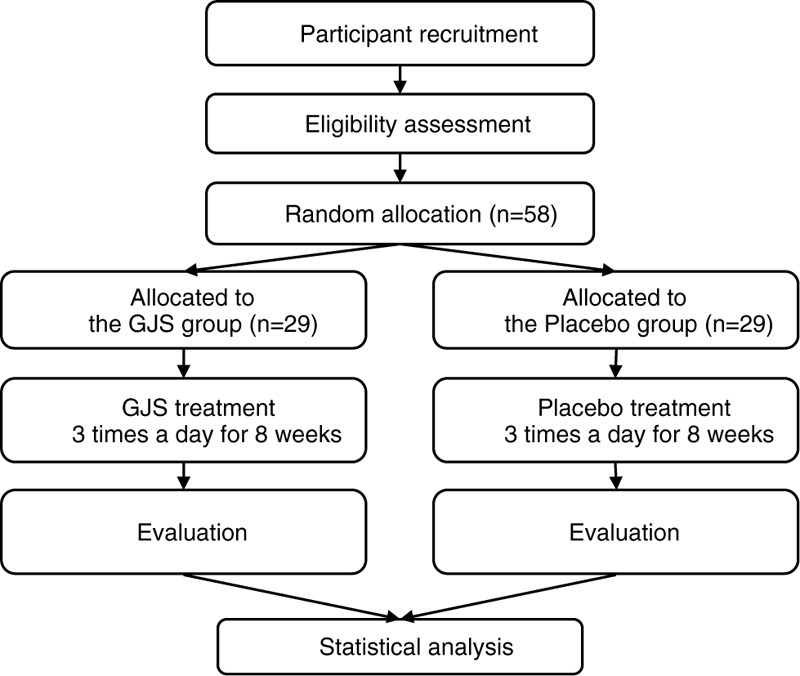
Study design flowchart. GJS = Gwakhyangjeonggi-san.

This randomized, double-blind (patient, practitioner, and assessor), placebo-controlled, parallel-group, clinical trial will be conducted at the Kyung Hee University Korean Medicine Hospital at Gangdong (Seoul, Republic of Korea). A total of 58 participants will be recruited for this trial and participants will be assigned either GJS treatment group or placebo controlled group. The trial will consist of an 8-week oral administration of GJS with 2 visits at 4-week intervals. Before enrollment, all participants will undergo a 7-day run-in period. The enrolled participants will be randomly allocated to 2 parallel groups: the GJS group and the placebo group. The study design flow chart is shown in Fig. 1 and Table 1. This protocol is based on the Standard Protocol Items: Recommendations for Interventional Trials.

**Table 1:**
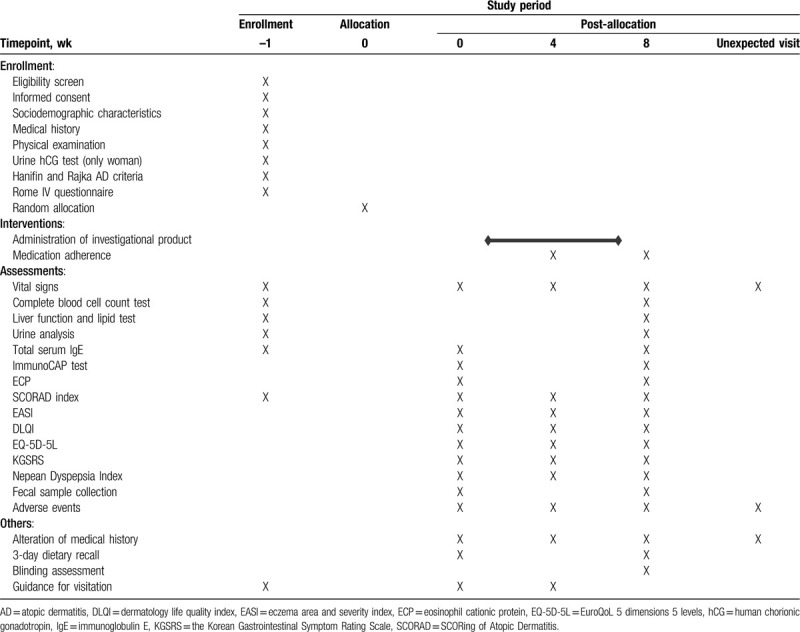
Schedule of enrollment, interventions, and outcome measurements for a randomized controlled trial assessing the safety and clinical efficacy of *Gwakhyangjeonggi-san* (GJS) on moderate atopic dermatitis with gastrointestinal symptoms.

### 2.3 Participants

#### 2.3.1 Inclusion criteria

Participants will be considered eligible for inclusion if they meet the following criteria:

1. Men or women aged 19 to 60 years.
2. Diagnosis of AD based on the Hanifin and Rajka diagnostic criteria, which require that ≥3 major features and ≥3 minor features be fulfilled.
3. Diagnosis of gastroduodenal or bowel disorder based on the ROME IV Questionnaire.
4. Moderate AD diagnosed by the SCORing of Atopic Dermatitis (SCORAD) index (score 25–50).
5. Ability to comprehend the purpose of the study, the study procedure, and the properties of the investigational drug, as well as voluntary agreement to participate in the study (provision of written informed consent).

#### 2.3.2 Exclusion criteria

The exclusion criteria will be as follows:

1. Diagnosis of concomitant skin disorders that make it difficult to accurately evaluate cutaneous symptoms of AD.
2. Use of orally administered corticosteroids or antibiotics, administration of psoralen and ultraviolet A therapy or immune-suppressants within the last 4 weeks preceding screening visit.
3. Use of medications including herbal preparations, dietary supplements, or probiotics for symptomatic improvement of AD within the last 4 weeks of screening.
4. Diagnosis of serious medical conditions that could interfere with clinical trial participation, including the following:
  4-1. Uncontrolled hypertension (systolic blood pressure ≥160 mmHg and diastolic blood pressure ≥100 mmHg).
  4-2. Uncontrolled diabetes mellitus (HbA1c ≥6.5%).
  4-3. Abnormal kidney function test results showing serum creatinine levels >2-fold the upper limit of the reference range.
  4-4. Abnormal liver function test results showing serum alanine aminotransferase or aspartate aminotransferase levels >2-fold the upper limit of the reference range.
  4-5. Other serious inflammatory and systemic diseases.
  4-6. Past or present history of malignant tumors.
5. Genetic disorders, such as galactose intolerance, Lapp lactase deficiency, or glucose-galactose malabsorption.
6. Intake of other clinical trial drugs within a month preceding the start date of the investigational drugs for the present trial.
7. Pregnancy, planning a pregnancy, or breastfeeding.
8. Known history of hypersensitivity or allergic reaction to the investigational drug or its ingredients.
9. Communication difficulties that prevent adequate compliance with the investigator's instructions.
10. Ineligibility for participation determined by the investigator.

#### 2.3.3 Withdrawal criteria

1. Participants’ withdrawal of consent.
2. Detection of eligibility violations.
3. Occurrence of a serious adverse event.
4. Use of prohibited medication.
5. Pregnancy.
6. Protocol violations during the study.
7. Total medication compliance under 80%.
8. Discontinuity of contact.
9. Unsuitability as judged by the investigators.

### 2.4 Sample size

No clinical trial has been conducted to evaluate the efficacy of GJS compared with placebo on the SCORAD index for AD. Therefore, we determined a required sample size of 23 per group based on recommendations by a previous statistical study.^[17]^ Considering an assumed dropout rate of 20%, 58 participants, 29 in each group will be required.

### 2.5 Recruitment

Recruitment began in September 2019 and is expected to end by October 2020. The study will be advertised through the bulletin boards at the hospital and local landmarks, the homepage of the hospital, the local newspaper, local online communities, and on public transportation. The posters will contain brief descriptions of the inclusion and exclusion criteria, the purpose of the study, and interventions used.

### 2.6 Randomization and blinding

Participants will be randomly assigned to 1 of the 2 groups with a 1:1 ratio. An independent statistician will generate the blocked randomization using SAS software version 9.4 (SAS institute. Inc., Cary, NC) and pass it to the pharmaceutical company (Hanpoong Pharm & Foods Co., Ltd.). Experimental drugs will be allocated and labeled based on the randomization number by the pharmaceutical company. Accordingly, the hospital will be provided with GJS and the placebo sealed in opaque aluminum packages and labeled with the random numbers. The participants will be assigned a randomization number and the clinical trial pharmacist will provide the packaged drugs to the participants based on the randomization number. All researchers and participants will be blinded for the assignment process until the trial is completed unless a serious adverse event occurs. The generated numbers will be sealed in opaque envelopes and stored in double-locked cabinets.

### 2.7 Intervention

Hanpoong Pharm & Foods Co., Ltd. manufactures the GJS and placebo in compliance with Korea Good Manufacturing Practice (KGMP) standards. Quality control and quality assurance for quality and safety testing, packaging, and contents of the treatment drug, including checks for potential contaminants such as heavy metals or steroids, will be undertaken by the manufacturers to ensure drug stability and quality.

GJS used in this study (Hangpoong *Gwakhyangjeonggi-san* extract granule) is dried, brown granules extracted with water. The extract is permitted and regulated by the MFDS and is composed of 13 herbs (Table 2). The placebo is made of lactose hydrate, corn starch, ginseng flavored powder, and caramel coloring. Both drugs will be identical in appearance, shape, taste, and color (brown).

**Table 2:**
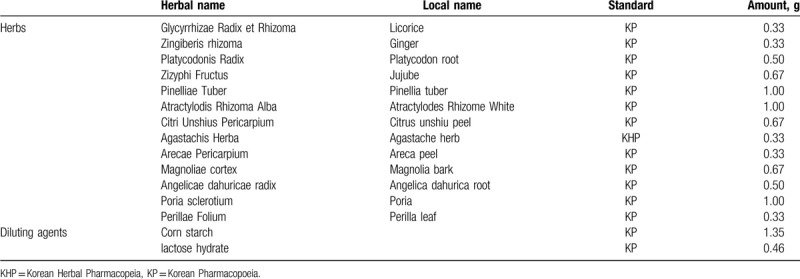
Composition of the *Gwakhyangjeonggi-san* (GJS).

GJS and placebo granules are sealed in opaque aluminum bags and administered to participants in doses of 3 g. The pharmacists will instruct the participants to dissolve GJS or placebo from each package in water and take the solutions 3 times per day 30 minutes before breakfast, lunch, and dinner or between meals for 8 weeks. All participants will be requested to return remains of drugs for calculating compliance.

Participants who have been receiving medication for chronic diseases such as hypertension and diabetes mellitus prior to the initiation of this study will be allowed to continue their regimen if it is a medication unlikely to affect the results. Care will be taken not to alter the dosage or type of drug during the study. Participants of both groups will be prohibited from the concomitant use of medication such as steroids, antihistamines, and immunosuppressive drugs that can potentially affect the outcome of this experiment until after the 8-week follow-up.

### 2.8 Outcome measurement

#### 2.8.1 Atopic dermatitis symptoms

Disease severity and quality of life will be assessed using SCORAD, the eczema area and severity index (EASI), the Dermatology Life Quality Index (DLQI), and EuroQoL 5 dimensions 5 levels (EQ-5D-5L), respectively. The primary outcome of the present study is the difference between the SCORAD of the 2 study groups before (week 0) and after (week 8) medication has been administered. SCORAD, EASI, DLQI, and EQ-5D-5L also will be assessed at week 0, 4, and 8.

#### 2.8.2 Gastrointestinal symptoms

The Korean Gastrointestinal Symptom Rating Scale (KGSRS) and Nepean Dyspepsia Index-Korean version (NDI-K) will be assessed at weeks 0, 4, and 8.

#### 2.8.3 Immune biomarker analysis

Total serum immunoglobulin E (IgE) and eosinophil cationic protein (ECP) will be evaluated at weeks 0 and 8.

The specific IgE levels for the following 12 specific antigens will be measured using the ImmunoCAP assay at weeks 0, and 8: egg white, milk, walnut, wheat, peanut, soy, shrimp, buckwheat, fish (cod), meat mixture, *Dermatophagoides pteronyssinus*, and *Dermatophagoides farina*. A blood sample will be obtained for further analysis of the immune biomarkers and metabolites.

#### 2.8.4 Microbiome analysis

Microbiome analysis will be conducted from fecal samples. The deoxyribonucleic acid (DNA) in the collected fecal samples are isolated using the FastDNA SPIN kit (MPBio). Polymerase chain reaction (PCR) will be performed using a primer to amplify the V3–V4 gene region of 16S ribosomal ribonucleic acid (rRNA) from the separated DNA. After purification of the amplified product, a genomic DNA library will be constructed and the nucleotide sequence analyzed. Fecal sample collection for microbiome analysis will be performed at week 0 and 8.

#### 2.8.5 Safety outcomes

For the safety of the study participants, complete blood cell counts, liver function tests, lipid tests, and urine analyses will be performed at the screening phase and end of the trial. Vital signs will be examined at every visit. For female participants, urine human chorionic gonadotropin (hCG) will be checked at the screening visit to test for pregnancy. The investigator will ask about any expected or unexpected adverse events and record details of adverse events in the case report form (CRF). When serious adverse events occur, the investigator will immediately report this to the institutional review board (IRB) within 24 hours. If a serious adverse event occurs, the investigators will determine whether it is acceptable, whether the experimental drug should be continued or stopped, and whether the trial should be amended or ended.

### 2.9 Data and safety monitoring

To protect the rights and welfare of the participants and to control the quality of the study, monitoring will be performed. According to the planned protocol and standard operating procedures, a clinical research associate (CRA) will monitor if the trials are proceeding according to the protocol by performing a cross-check of the informed consent form, CRF, original chart of the participants, and drug management records. The trial data will be saved in an electronic data capture system (Medidata Rave; Medidata Solutions Inc., New York, NY). A clinical research coordinator will call patients during weeks 2 and 6 to promote participant retention and drug compliance. A separate storage location will be used to store the data and documents related to the clinical trial, and security will be maintained. After drafting the final report, a person in charge of the storage will be designated to keep the documents related to the clinical trial for 3 years from completion of the clinical trial. After completion of the study, the originals of the clinical trial CRF and other data collected and acquired during the study will be stored separately in accordance with the IRB regulations.

### 2.10 Statistical analysis

The measured variables, including the primary and secondary outcomes, will be assessed using the full analysis set based on the intention-to-treat principles. The per-protocol analysis set will be used for the sensitivity analysis. The baseline characteristic values in both groups will be expressed as a mean and standard deviation for continuous variables that are normally distributed or medians and interquartile ranges for non-normal data. Frequencies and percentages will be used to present categorical variables. Baseline differences between groups will be assessed using an independent 2-sample *t* test or Wilcoxon rank-sum test for continuous variables. The Chi-square test or Fisher exact test will be used for categorical variables. A mixed-effect model repeated measure (MMRM), which sets participants as a random factor and the group and visit as fixed factors, will be performed to analyze the between-group differences in the primary and secondary outcome measures. The Student paired *t* test and repeated-measures analysis of variance (ANOVA) will be used to evaluate the intra-group time changes in continuous values. Chi-square or Fisher exact tests will be used to evaluate the intra-group time changes in categorical values. MMRM does not require the replacement of missing data, and the missing data in the intra-group analysis will be replaced using the last observation carried forward method. The level of significance will be set at 0.05 (2-tailed). All analyses will be performed by an independent statistician using SAS version 9.4 (SAS Institute Inc., Cary, NC).

### 2.11 Ethics and dissemination

This study was approved by the IRB of Kyung Hee University Korean Medicine Hospital at Gangdong (KHNMCOH2019-06-002-001, version 1.4, 11-SEP-2019). This trial has been registered at the Korean National Clinical Trial Registry, Clinical Research Information Service (KCT0004299) before the recruitment, which is a primary registry of the World Health Organization International Clinical Trials Registry Platform. This study was also approved by the MFDS for the investigational new drug (IND) tract. Any protocol modifications will be approved by the IRB of the Kyung Hee University Korean Medicine Hospital at Gangdong and MFDS.

The trial will be performed in compliance with the Declaration of Helsinki, and according to Good Clinical Practices as described by the MFDS. Written informed consent will be obtained by the investigator from all participants prior to enrollment. The written informed consent form will include information on the background and purpose of the study, experimental and placebo drugs, outcome measures, and possible benefits and harms. The participants will be immediately notified when new facts regarding the study are found. The investigator will explain the study in non-scientific language. All participants will be given enough time to decide whether they wish to participate in the trial. The confidentiality of their personal information will be protected. Throughout and subsequent to the trial, all documents and data will remain secure in a locked cabinet or as password-protected computer files.

Investigator, sponsor, CRA, and IRB members associated with this study can access the records of the participants for the purpose of managing the monitoring, testing, and progress of this study. And auditor from the MFDS can access the records in order to audit whether the trial was conducted in accordance with law and regulation. All documents, including the CRF, must be recorded by the participant's identification code (generally the participant's initials) and not the participants’ name.

## 3 Discussion

AD is a long-standing inflammatory skin disease. Therefore, it is necessary to develop medicines that have no adverse effects when used for long-term therapy. Conventional treatment for AD has been focused on directly reducing the inflammation. However, there have been many limitations of such treatment with adverse effects after long-term use, symptoms relapse after the discontinuation of medication, and refractory cases for medications.

Extensive research has investigated the interaction of the gut microbiota with nutrition, metabolism, physiology, and immune function. Many reports have identified a link between microbiome and many chronic conditions such as obesity, inflammatory bowel diseases, diabetes mellitus, allergic rhinitis, and AD.^[18–22]^ Selective modulation of host-microbiome using pro-, pre-, synbiotics to treat AD has been of particular interest to many investigators.^[23]^

For many decades, many herbal drugs have demonstrated their efficacy and safety in treating AD. A Cochrane systematic review of 28 studies involving 2306 participants found that an orally administered herbal drug could improve the quality of life in children with AD.^[24]^ However, further well-designed clinical studies are needed to determine the efficacy of herbal drugs.

An increasing number of studies have demonstrated that the therapeutic effect mechanism of herbal drugs is linked with gut microbiota.^[25]^ Many clinical studies that have reported the efficacy of herbal formulas in treating AD have observed skin symptoms using SCORAD and EASI and assessed the modulation of immune biomarkers such as serum IgE, eosinophil counts.^[24]^ To date, no clinical studies have evaluated the efficacy of herbal drugs on gut microbiota in patients with AD. In the present study, GJS will be administered for 8 weeks. Its impact on skin symptoms, systemic immune biomarkers, and gut microbiota will be evaluated.

To our knowledge, this is the first clinical study to investigate the efficacy of GJS for patients with AD, as well as the first study to investigate the efficacy of herbal drugs on gut microbiome. This study will provide evidence regarding the use of GJS in treating AD.

## Acknowledgment

The authors thank So Young Jung and Bo-Young Kim at the Korea Institute of Oriental Medicine for monitoring trial and developing electronic CRF.

## Author contributions

**Conceptualization:** Mi Ju Son, Jeeyoun Jung.

**Funding acquisition:** Jeeyoun Jung.

**Investigation:** Min Hee Kim, Minseo Kang.

**Methodology:** Young-Eun Kim.

**Project administration:** Inhwa Choi.

**Supervision:** Jeeyoun Jung, Inhwa Choi.

**Writing – original draft:** Mi Ju Son, Min Hee Kim.

**Writing – review & editing:** Minseo Kang, Young-Eun Kim, Jeeyoun Jung, Inhwa Choi.
